# Data for global lysine-acetylation analysis in rice (*Oryza sativa*)

**DOI:** 10.1016/j.dib.2016.02.032

**Published:** 2016-02-20

**Authors:** Yehui Xiong, Kai Zhang, Zhongyi Cheng, Guo-Liang Wang, Wende Liu

**Affiliations:** a State Key Laboratory for Biology of Plant Diseases and Insect Pests, Institute of Plant Protection, Chinese Academy of Agricultural Sciences, Beijing 100193, China; b Jingjie PTM BioLab (Hangzhou) Co. Ltd., Hangzhou 310018, China; c Department of Plant Pathology, Ohio State University, Columbus 43210, OH, USA

**Keywords:** Rice, Acetylation, LC–MS/MS

## Abstract

Rice is one of the most important crops for human consumption and is a staple food for over half of the world׳s population (Yu et al., 2002) [1]. A systematic identification of the lysine acetylome was performed by our research (Xiong et al., 2016) [2]. Rice plant samples were collected from 5 weeks old seedlings (*Oryza sativa*, Nipponbare). After the trypsin digestion and immunoaffinity precipitation, LC–MS/MS approach was used to identify acetylated peptides. After the collected MS/MS data procession and GO annotation, the InterProScan was used to annotate protein domain. Subcellular localization of the identified acetylated proteins was predicted by WoLF PSORT. The KEGG pathway database was used to annotate identified acetylated protein interactions, reactions, and relations. The data, supplied in this article, are related to “A comprehensive catalog of the lysine-acetylation targets in rice (*O. sativa*) based on proteomic analyses” by Xiong et al. (2016) [2].

**Specifications Table**

**Table:** 

Subject area	*Plant Biology*
More specific subject area	*Rice modification proteomics*
Type of data	*MS data, Tables, Figures*
How data was acquired	*Data-dependent acquisition of affinity purification and LC–MS/MS.*
Data format	*Raw and analyzed.*
Experimental factors	*Plants were harvested and washed with double distilled water, immediately frozen in liquid nitrogen and stored at* −80 °C *until protein extraction and following experiment.*
Experimental features	*The protein were extracted and digested with trypsin, peptides were enriched by antibody immunoaffinity and subjected to the LC–MS/MS analysis.*
Data source location	*State Key Laboratory for Biology of Plant Diseases and Insect Pests, Beijing, China*
Data accessibility	*1. Raw data is available on PRIDE database, accession ID number is PXD002291;*
*2. Analyzed data are supplied with this article.*

## Value of the data

• The data are useful for model plant/crop biology research and protein modification research in plant or animal.
• We used agarose conjugated with our high affinity pan anti-acetyl-lysine antibody for immunoaffinity enrichment, which improve the recognition range and efficiency.
• The number of lysine-acetylated sites in this data was 23-times greater and the number of acetylation proteins was 16-times greater than in the other previous report.

## 1 Data

As an important model plant for biological research, rice also is one of the most important crops and food for human consumption [1]. We performed a systematic identification of the lysine acetylome in rice, recently [2].

**Fig. 1:**
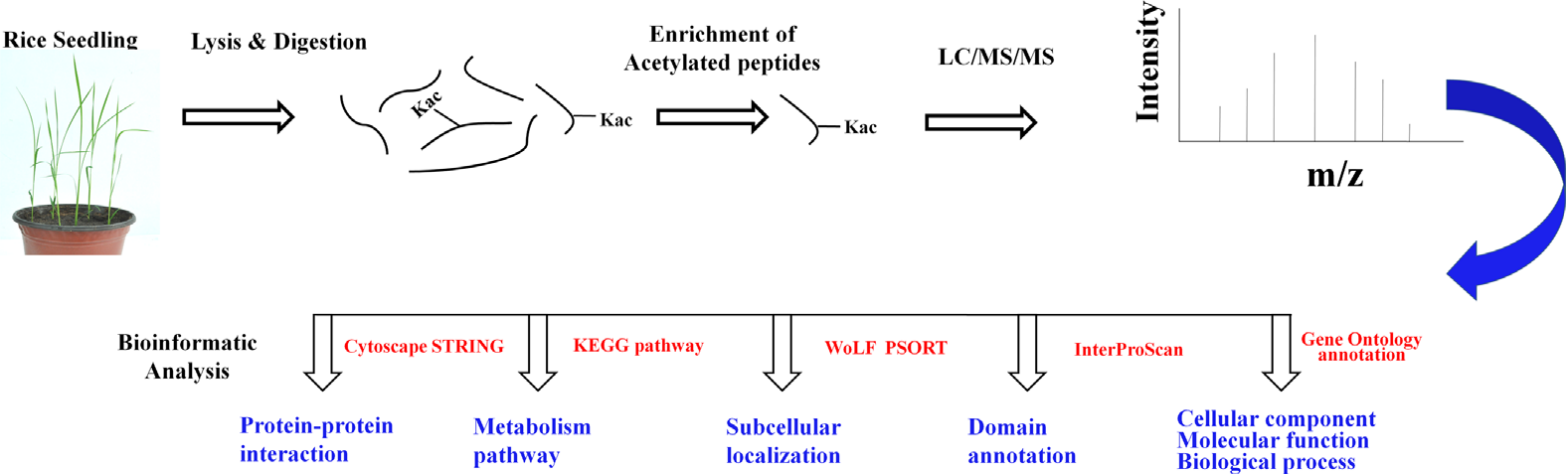
Experimental and bioinformatics workflow of the rice lysine acetylome analysis.

**Fig. 2:**
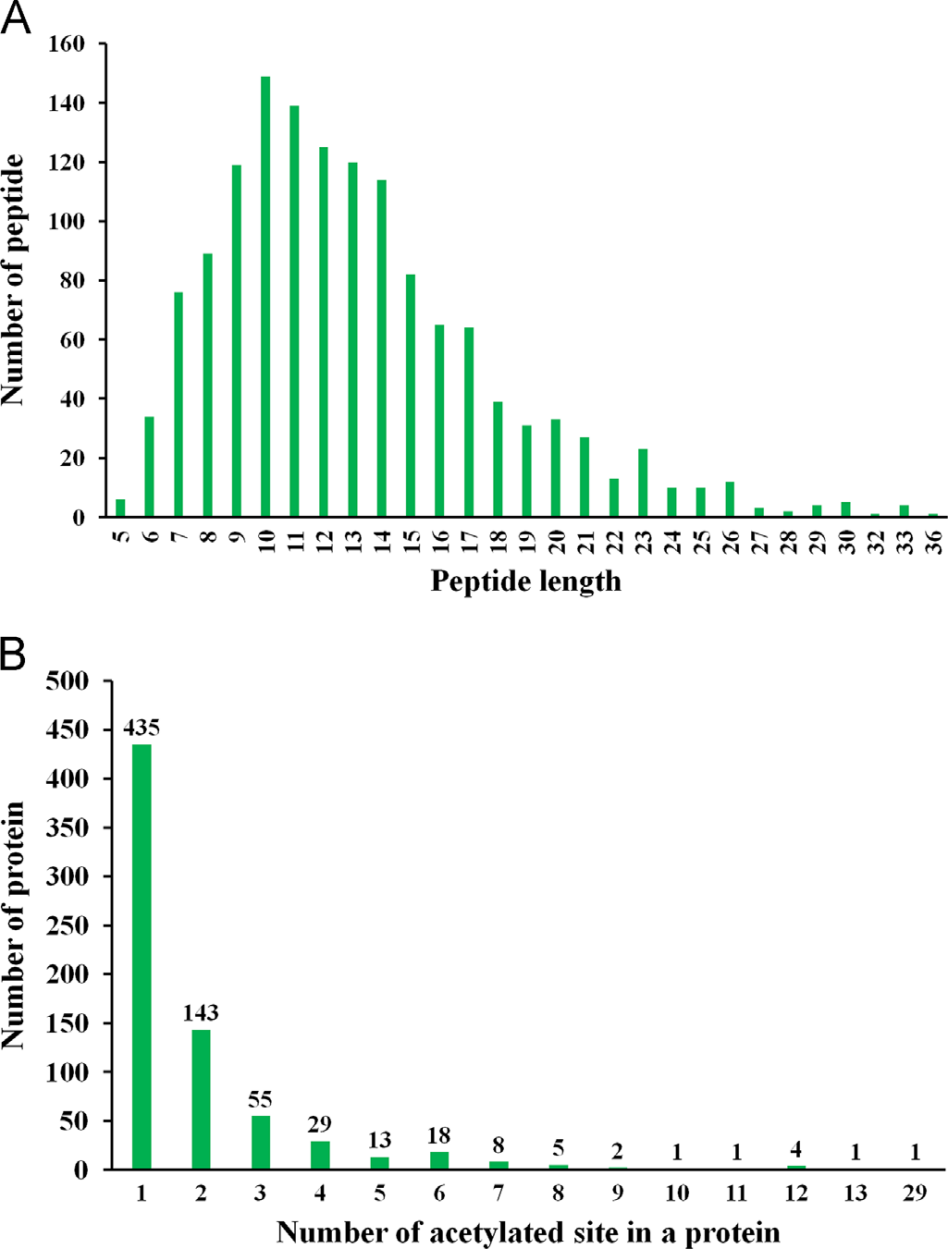
Distribution of acetylated peptides based on their (A) length and on their (B) number of acetylation peptides.

**Fig. 3:**
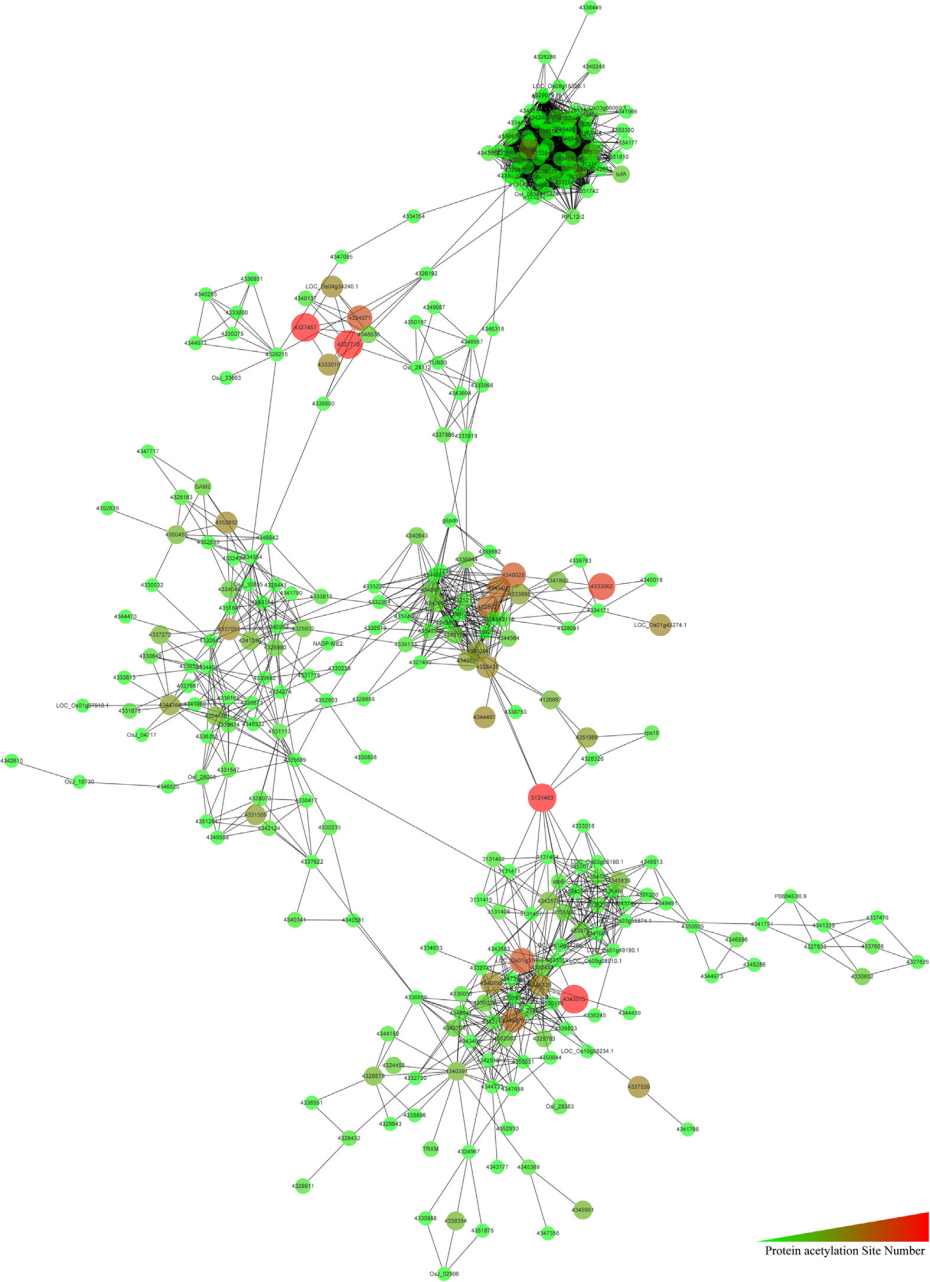
Interaction networks of acetylated proteins in rice.

Fig. 1 show the experimental and bioinformatics workflow of the rice lysine acetylome. A total of 1337 lysine-acetylation (Kac) sites on 716 proteins were identified, and data is presented in Supplementary Table S1. The raw data were available on PRIDE database with accession number PXD002291. Most of the peptides contain 7–15 amino acids (Fig. 2A). Of the 716 proteins, 435, 143, and 55 contained 1, 2, and 3 Kac sites, respectively (Fig. 2B). The information on the Kac peptides and proteins were summarized in Table S1. Kac motif is listed in Supplementary Table S2. To better understand the global cellular functions of the acetylated proteins in rice, a GO functional classification was conducted in terms of their molecular functions, cellular components, and biological processes, the GO data is listed in Supplementary Table S3. KEGG also conducted and the detail data listed in Supplementary Table S4. We also used Cytoscape software to generate an interaction network for all of the acetylated proteins. When the threshold confidence score was set at 0.70, associations were detected among 347 proteins (Supplementary Table S5). As shown in Fig. 3, this network contained a number of acetylated proteins at key hubs. The detailed data were discussed by Xiong et al. [2]. The raw data about this research is available on PRIDE database, accession number is PXD002291.

## 2 Experimental design, materials and methods

### 2.1 Rice plants and growth conditions

Rice (*Oryza sativa*, Nipponbare) seeds were sterilized with 75% ethanol, 1 min; 40% NaClO, 30 min; followed with double distilled water washing 3 times; and transferred to half-strength Murashige and Skoog medium [3]. The rice seedlings were then transplanted into soil and kept in a growth chamber at 25 °C with 80% relative humidity and a 16-h-day/8-h-night cycle. After 5 weeks growth, the whole rice plants including leaves, stems, and roots (two independent, whole rice plant samples) were collected and washed with double distilled water for further LC–MS/MS analysis.

### 2.2 Protein extraction and peptide digestion

The protein extraction and peptide digestion procedures were modified from a previous report [4]. Briefly, the whole rice seedlings were ground in liquid nitrogen and then supplemented with 8 M urea containing 1 mM DTT, 2 mM EDTA, 1% Protease Inhibitor Cocktail Set III (Calbiochem), and HDAC inhibitor (50 mM sodium butyrate, 30 mM nicotinamide, 3 uM trichostatin A). The sample was then sonicated with 12 short bursts, 10 s per burst and separated by 30-s cooling intervals. The preparations were centrifuged at 4 °C for 10 min at 20,000*g* to remove unbroken cells and debris, the 2-D Quant kit (GE Healthcare) was used to determine the protein concentration of the preparation. Proteins were then precipitated with 15% trichloroacetic acid overnight at 4 °C. The precipitate was washed three times with ice-cold acetone and air-dried at 4 °C.

For protein digestion, 100 mM NH_4_HCO_3_ was added, which was followed by the addition of trypsin (Promega) at an enzyme-to-substrate ratio of 1:50; the digestion was performed at 37 °C for 12 h. Reduction and alkylation were performed by adding 5 mM dithiothreitol (DTT) at 56 °C for 45 min and 15 mM iodoacetamide (IAM) for 30 min at room temperature in darkness. The reaction was quenched with 30 mM cysteine at room temperature for 20 min. Then, additional trypsin at an enzyme-to-substrate ratio of 1:100 was added, and the preparation was incubated at 37 °C for 4 h to ensure complete digestion. The final digest was lyophilized in a SpeedVac and stored at −80 °C.

### 2.3 Immunoaffinity precipitation

The Kac peptides immunoaffinity enrichment was perform as described [4], [5]. Briefly, the tryptic peptides for Kac enrichment were re-dissolved in NETN buffer (100 mM NaCl, 1 mM EDTA, 50 mM Tris, 0.5% Nonidet P-40, pH 8.0) before anti-acetyl-lysine agarose beads (catalog no. PTM-104, PTM Biolabs) were added; the mixture was incubated at 4 °C with gentle shaking overnight. The agarose beads were then gently washed three times with NETN buffer, twice with ETN buffer (100 mM NaCl, 1 mM EDTA, 50 mM Tris, pH 8.0), and once with pure water. Finally, the bound peptides were eluted from the agarose beads with 1% trifluoroacetic acid (TFA) and were lyophilized to dryness in a SpeedVac. The resulting peptides were desalted with C18 ZipTips (Millipore) before LC–MS/MS analysis.

### 2.4 LC–MS/MS

LC–MS/MS has been extensively used to identify modified peptides [4], [5], [6], [7]. We re-dissolved the peptides of each sample in 6 μL of mobile phase A (2% CAN/0.1% FA) and then loaded the preparation onto a trap column (Acclaim PepMap 100 C18, 75 μm DI, 2 cm length, Dionex) by EASY nLC1000 nanoUPLCsystem (ThermoScientific). For separation, the peptide was eluted onto a C18 analytical column (Acclaim PepMapRSLC, 50 μm DI, 15 cm length, Dionex). A 40-min gradient was run at 280 nL/min, starting from 5 to 30% mobile phase B (98% ACN, 1.9% H_2_O, 0.1% FA) for 30 min, 30–40% mobile phase B for 4 min, 40–80% mobile phase B for 2 min, and 80% mobile phase B for 4 min.

The peptides were injected into an NSI source coupled to a tandem mass spectrometry (MS/MS) in Orbitrap Q Exactive (ThermoScientific). The electrospray voltage applied to the NSI source was set at 1.8 kV. The parent ions of peptides were detected in the Orbitrap for full-MS scan; the resolution was set at 70,000, and the *m*/*z* scan range was 350–1800. The top 15 peptides were the selected for the following MS/MS fragmentation in the Orbitrap by using HCD at 28% NCE with 4% stepped NCE, and the resolution for ion fragments was set at 17,500. The MS/MS threshold ion count was set at 4×10^4^ with 2.5-s dynamic exclusion. For generation of MS/MS spectra, 2×10^5^ ions were accumulated.

### 2.5 Database search

The collected MS/MS data were processed by MaxQuant with the integrated Andromeda search engine (version 1.4.1.2), Mascot research engine (version 2.3), and pFind (version 2.8) against the Uniprot_OS Japonica Database, concatenated with the reverse decoy database. Trypsin/P was specified as the cleavage enzyme, the maximum missing cleavage was set at 3, and up to 4 modifications and 5 charges were allowed in each peptide. Mass tolerance was set at 5 ppm for precursor ions and 0.02 Da for fragment ions. Carbamidomethylation on Cys was specified as the fixed modification, and oxidation on Met, acetylation on Lys, and acetylation on protein N-terminal were specified as variable modifications. The false discovery rates (FDRs) for protein, peptide, and modification site were all specified as 0.01. The minimum peptide length was set at 7. To obtain high-confident results, Lys-acetylation sites with localization probability <0.75 or from reverse protein sequences were removed.

### 2.6 Acetylated protein annotation

GO annotation of identified acetylated proteins were derived from ID mapping data of the UniProt database (ftp://ftp.uniprot.org/pub/databases/uniprot/current_release/knowledgebase/idmapping/) [8]. GO information for each protein was extracted from the ID mapping data as UniPortKB protein accession number. The KEGG pathway database was used to annotate identified acetylated protein interactions, reactions, and relations. The sequence of identified acetylated protein were used as input data for KOALA (KEGG Orthology and Links Annotation) software, and *O. sativa* japonica (taxonomy number: 39947) was set as the background species for search. Each input protein was then annotated by K number to a given sequence data set for subsequent analyses with reconstruct pathway [9]. InterProScan, a software package that allows protein and nucleic sequences to be scanned against InterPro׳s signatures, was used to annotate protein domain from the InterPro database, which combines signatures from multiple, diverse databases into a single searchable resource [10]. WoLF PSORT was used to predict the subcellular localization of the identified acetylated proteins [11].
